# Electrochemical Behavior of Neratinib at a Boron-Doped Diamond Electrode: Role of Tween 20 in Interfacial Signal Enhancement

**DOI:** 10.1021/acsomega.6c05232

**Published:** 2026-09-01

**Authors:** Gülsüm Ece Meraki, Hülya Silah, Bengi Uslu

**Affiliations:** † Faculty of Pharmacy, Department of Analytical Chemistry, 37504 Ankara University, Ankara 06560, Turkiye; ‡ The Graduate School of Health Sciences, Ankara University, Ankara 06110, Turkiye; § Faculty of Science, Department of Chemistry, 121945 Bilecik Seyh Edebali University, Bilecik 11100, Turkiye; ∥ Integrated Research Center (BUTAM), Ankara University, Yapracik, Etimesgut, Ankara 06790, Turkiye

## Abstract

Neratinib (NER) is an irreversible tyrosine kinase inhibitor employed in the therapy of HER2-positive breast cancer. In the present research, the electrochemical oxidation of NER at a boron-doped diamond electrode (BDDE) was surveyed with particular emphasis on the act of surfactant-mediated interfacial influences. Cyclic and differential pulse voltammetry were used to characterize the oxidation behavior of NER and to establish an analytical approach for its determination. Among the explored anionic, cationic, and nonionic surfactant species, Tween 20 produced the most proper electrochemical reply, obviously augmenting the anodic peak current and improving signal stability. Electrochemical impedance spectroscopy (EIS) further represented that Tween 20 considerably revised the electrolyte/BDDE interface, increasing the charge-transfer resistance of the ferri/ferrocyanide redox probe from 510 to 1822 Ω. Importantly, this increment in interfacial resistance comprised concurrently with enhancement of the NER voltammetric response, indicating that Tween 20 does not act through a nonspecific acceleration of electron-transfer kinetics. Conversely, the monitored signal enhancement is consistent with affirmative NER–surfactant interplays and a modified interfacial microenvironment that assists the electrochemical accessibility of NER. Scan-rate and pH-dependent investigations defined that NER undergoes an irreversible, predominantly diffusion-controlled oxidation involving proton-coupled electron transfer. Under the optimized experimental conditions at pH 7.02 containing 100 μM Tween 20, the DPV signal was linear the NER concentration between 0.03–1.50 μM (16.7–836 ng mL^–1^). The limits of detection and quantification were 5.09 nM (2.84 ng mL^–1^) and 15.16 nM (8.44 ng mL^–1^), respectively. Overall, the outcomes demonstrate that the analytical enhancement of NER at BDDE originates from surfactant-mediated, analyte-specific interfacial impacts and ensure a mechanistic basis for its sensitive voltammetric determination.

## Graphical abstract

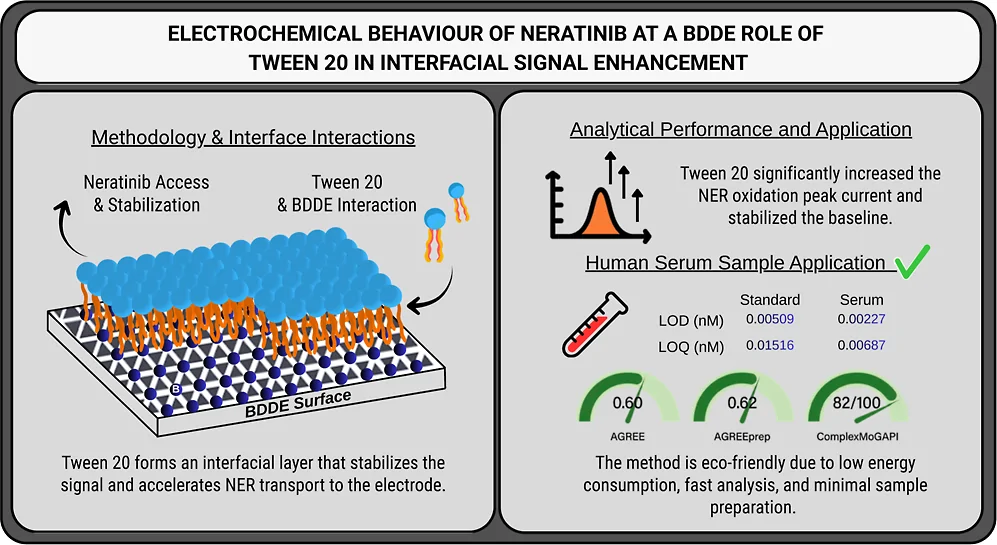

## 1 Introduction

Breast cancer is a worldwide health problem as one the foremost causes of death in women, standing as the world’s second most frequently diagnosed malignancy. According to WHO data released in 2020, the number of deaths attributed to breast cancer was approximately 685,000 worldwide. In recent years, therapeutic alternatives to breast cancer treatments have continued to introduce a wide selection of novel pharmacological agents.^1,2^ In this context the Human Epidermal Growth Factor Receptor 2 (ErbB2/HER2) is acknowledged as an indispensable therapeutic target. Elevated HER2 levels are detected in nearly one-third of breast cancer cases, and this is strongly associated with heightened tumor aggressiveness and an overall poor clinical prognosis.^3–5^

NER is an orally available small-molecule tyrosine kinase inhibitor that acts through irreversible inhibition of its target receptors that targets multiple HER variants. Preclinical studies have indicated that NER effectively suppresses HER2-positive cell proliferation by inducing cell-cycle arrest. Clinically, NER (commercially available as Nerlynx) has received regulatory ratification from the U.S. Food and Drug Administration (FDA) as an extended adjuvant monotherapy for patients with early stage HER2-positive breast cancer patients that were treated with trastuzumab-based adjuvant.^6,7^

Common methodologies to determine NER mostly include ultra performance liquid chromatography/mass spectrometry system (UPLC-MS/MS) and spectrofluorimetry.^8–11^ The established chromatographic techniques are usually trusted at the determination and quantification of drug molecules.^12^ However, these chromatographic techniques typically suppose complicated, often necessitate multiple sample-treatment steps, increasing both workload and analysis time steps. Additionally, its equipment is relatively expensive and requires trained personnel. Since it requires a controlled laboratory environment, it appears to be impossible to miniaturize as a portable system.^13^ Additionally, minor deviations in exposure levels can affect the desired therapeutic efficacy negatively or increase the risk of its side effects. Consequently, reliable, and sensitive analytical determination methods are required to ensure the precise measurement of these anticancer agents, particularly at low concentration levels related to pharmacokinetic monitoring. Electroanalytical approaches offer practical advantages by providing rapid and cost-effective measurements with minimal sample preparation effort.

A BDDE (Boron-doped diamond electrode) is prepared by embedding the boron element into the bulk diamond material. Since their introduction in 1992, BDDEs were utilized extensively across multiple areas of electrochemistry. This electrode variant has multiple distinctive features, such as a wide potential window; reaching up to approximately 3.5 V in aqueous media depending on boron doping and surface conditioning, along with high overpotentials for gas evolution and carbon corrosion, minimal background currents, and chemical durability.^14,15^ Additionally, their inert surfaces and very low sensitivity to contamination and adsorption processes make them favorable electrodes for the electrochemical analysis of various pharmaceutically active substances that require high oxidation potential.^16,17^ Furthermore, the usage of an unmodified BDDE permits direct electrochemical measurements without needing permanent functionalization of surface, biological recognition elements, or nanomaterial coatings.

A possible strategy for modulating electrochemical replies without persistent electrode modification comprises the incorporation of surfactants directly into the supporting electrolyte. Because of their low cost, wide availability, high stability, and minimal environmental impact, surfactants are widely employed in electrochemical sensing applications for analyte detection. Beyond conventional electrode surface modification strategies involving nanomaterials or other functional coatings, the incorporation of surfactants directly into the electrolyte solution offers an alternative and effective approach to improve the sensitivity/selectivity of voltammetric techniques. Surfactant molecules can get adsorbed at the solution–electrode interface, thereby inducing in situ modifications of the electrode surface, positively affecting its electrochemical behavior.^18^ In addition, the solubilization of electroactive compounds within micellar aggregates can significantly regulate the properties of electrode surfaces, thereby influencing the electrochemical behavior. Especially, it is well-known that the interaction between its aggregates and solutes in the solution phase are controlled by the diffusion process.^19^

Surfactants are amphiphilic molecules that make the aggregate formation and accumulation at its electrode interfaces possible (gas–water, oil–water, or electrode–solution), because of their hydrophilic head and hydrophobic tail groups. Surfactant molecules can be categorized into anionic, cationic, nonionic, and zwitterionic groups according to the charge characteristics of hydrophilic head groups of them. Through adsorption and interfacial organization abilities, surfactants can alter electrode surface properties, regulate their mass transport and electron transfer kinetics.^20^

Critical micelle concentration (CMC) is a fundamental parameter that determines the behavior of a surfactant. Above the CMC, surfactant molecules self-assemble into micellar structures in solution. Micelle formation can facilitate the solubilization, preconcentration, and stabilization of hydrophobic analytes near the electrode/electrolyte interface, whereas below the CMC, surfactant molecules are predominantly adsorbed at the electrode/electrolyte interface. Consequently, the concentration of the surfactant relative to its CMC plays a crucial role in determining its electrochemical effect.^21–23^ In this context, Tween 20, a nonionic surfactant, is particularly advantageous due to its ability to form stable interfacial layers and provide a favorable microenvironment for hydrophobic drug molecules.^24,25^

Nevertheless, restricted knowledge is obtainable on how surfactant molecules impact the electrochemical oxidation of NER at surface of BDDE. Especially, the relationship between surfactant-stimulated interfacial alterations and the voltammetric reply of NER remains to be clarified. Examinations of these results may supply further comprehension into the interfacial processes governing NER oxidation and contribute to optimization of its electrochemical determination.

Accordingly, to the best of our information, this research describes for the first time the electrochemical examination of NER at an unmodified BDDE and systematically inspects the impact of surfactant species on voltammetric behavior of it. In contrast to many electrochemical sensing strategies that rely on complex surface modification or biological recognition elements such as enzymes or antibodies, the proposed approach enables direct electrochemical determination of NER without any additional modification. This feature simplifies the analysis procedure by reducing the preparation time and minimizes the inconsistencies associated with surface functionalization. Anionic (SDS), cationic (CTAB), also nonionic (Tween 20) surfactants were explored to make clear the effects of surfactant sort and concentration on the electrochemical reply of NER. CV and DPV, together with scan-rate and pH-dependent studies, were engaged to characterize the oxidation behavior of NER, while EIS was utilized to evaluate surfactant-stimulated alterations at the BDDE/electrolyte interface. The experimental electrochemical outcomes were further supported by computational calculations to obtain extra understanding into the suggested oxidation mechanism. Following mechanistic and interfacial optimization, the BDDE–Tween 20 system was used for the quantitative determination of NER and subsequently assessed in human serum samples. As a result, beyond establishing a sensitive voltammetric process, this research provisions the first realization into the influence of surfactant-mediated interfacial results on the electrochemical reply of NER at BDDE.

## 2 Materials and Methods

### 2.1 Reagents and Solution Preparation

Analytical-grade chemical reagents were exploited throughout the study. KCl, NaOH, NaCl, HCl, NaH_2_PO_4_·2H_2_O, Na_2_HPO_4_, H_3_BO_3_, K_3_[Fe­(CN)_6_], K_4_[Fe­(CN)_6_]·3H_2_O, H_3_PO_4_, CH_3_COOH, d-glucose, anhydrous caffeine, ascorbic acid, Tween 20, sodium dodecyl sulfate (SDS), and cetyltrimethylammonium bromide (CTAB) were supplied by Sigma-Aldrich. Dimethyl sulfoxide (DMSO) and CH_3_COONa·3H_2_O were acquired from ISOLAB and Riedel-de Haën, respectively. Analyte NER was supplied by MedChemExpress with analytical grade. Its IUPAC name is (2E)-*N*-[4-[[3-chloro-4-[(pyridin-2-yl)­methoxy]­phenyl]­amino]-3-cyano-7-ethoxyquinolin-6-yl]-4-(dimethylamino)­but-2-enamide. Its chemical structural is illustrated in Figure S1.

Millipore water purification system (Milli-Q model) ensured the deionized water utilized throughout solution preparation. pH of solution was measured and set employing an Orion SA 720 pH meter (USA). Electroanalytical studies were performed under ambient laboratory temperature conditions.

Stock solutions of NER at 1.0 × 10^–3^ M were prepared using DMSO as the solvent. To achieve the desired NER concentration for experimental analysis, the stock solution was diluted in the respective buffer solutions including acetate buffer solution (ABS), phosphate buffer solution (PBS), also britton robinson buffer (BRB). PBS was prepared using NaH_2_PO_4·_2H_2_O and Na_2_HPO_4_ (0.1 M, pH 6.02, 7.02, 8.02). ABS were prepared with CH_3_COOH and CH_3_COONa_·_3H_2_O (0.1 M, pH 5.7). BRB media spanning pH 2.0–12.0 were obtained from an equimolar mixture of phosphoric acid, acetic acid, and boric acid, each at a concentration of 40 mM, after which the pH was precisely adjusted to the desired value by the controlled addition of concentrated NaOH. The ferri/ferrocyanide redox couple, obtained from K_3_[Fe­(CN)_6_] and K_4_[Fe­(CN)_6_]·3H_2_O, served as the electrochemical probe for EIS measurements.

Prior to electrochemical analysis, commercially accessible human serum (Sigma-Aldrich), maintained at 253 K until application, was allowed to reach 298 K. A serum-based NER sample was prepared by combining 5.4 mL of DMSO with 3.6 mL of serum and subsequently spiking the mix with 1.0 mL of 1.0 × 10^–3^ M NER standard solution. Following centrifugation at 5000 rpm for 20 min, the outcoming supernatant was separated and propitiously diluted with PBS adjusted to pH 7.02. Aliquots of the diluted supernatant were then subjected to electroanalytical measurements.

### 2.2 Electrochemical Apparatus and Measurements

Electrochemical readings were performed with an Autolab PGSTAT204 workstation (Metrohm Autolab B.V., The Netherlands) using a ten mL electrochemical cell equipped with a three electrode configuration. A 3 mm diameter BDDE (D-534-SA, Windsor Scientific Ltd., UK) served as the indicator electrode, while an Ag/AgCl electrode containing 3 M KCl (BASi, USA) and a platinum wire (BASi, USA) functioned as the reference and counter electrodes, respectively. Before each mensuration, the BDDE was mechanically polished on a polishing pad making use of an alumina slurry to get a clean and reproducible electrode surface. Voltammetric experiments were performed at 25 ± 1 °C. The pH of the solutions was monitored using a SevenCompac pH/Ion S220 m (Mettler Toledo, Switzerland) equipped with a combination glass electrode.

The voltammetric response of NER was optimized by examining different supporting electrolyte media, pH conditions, and surfactant environments. The electrode process was then investigated by CV through scan rate studies. Under the optimized DPV conditions, a calibration curve was established and successfully applied to the determination of NER in commercial human serum samples. Method accuracy and reliability were evaluated using standard addition recovery experiment.

CV characterization of NER was accomplished in PBS (pH 7.02) over a potential scale extending from −0.1 to +1.5 V. To investigate the influence of scan rate (*v*), voltammograms were recorded from 10 to 1000 mV s^–1^. For quantitative measurements, DPV was used because suppression of capacitive and background charging contributions ensures improved sensitivity for analytical determination.^13^ The final DPV conditions consisted of a pulse time of 500 ms, pulse amplitude of 50 mV, scan rate of 20 mV s^–1^, and pulse width of 10 mV. Each experimental condition was assessed in triplicate, and the corresponding mean values were used for throughput analysis.

For impedance characterization, a 5 mM [Fe­(CN)_6_]^3–^/^4–^ redox couple prepared in 0.1 M KCl was implemented as the electrochemical probe. Impedance spectra were recorded at the open-circuit potential within the 100 kHz–0.1 Hz frequency domain using an AC perturbation of 10 mV and 50 frequency points distributed on a logarithmic scale. To keep away from variations in ionic strength during evaluation of the surfactant action, all surfactant-containing solutions were prepared handling the identical supporting electrolyte.

Computational computations were carried out for the isolated molecule in the gas phase. Gaussian 09 Rev. C.01 was used for the quantum-chemical calculations, whereas GaussView 5.0.9 was utilized for molecular structure construction and visualization.

## 3 Electrochemical Results and Discussion

### 3.1 Electrochemical Behavior of NER under Different Electrolyte and pH Conditions

Supporting electrolyte solutions are used to suppress the electrostatic migration of electroactive molecules toward the electrode surface, ensuring that mass transport is dominated by diffusion and yielding well-defined diffusion-controlled current responses.^26^ Most importantly, the pH of the buffer plays a decisive role in governing the electrochemical redox behavior of the target analyte. Additionally, investigating the electrochemical behavior of analyte across a range of pH values reveals the electron–proton coupling, and the reaction mechanism involved in the electrode process.^27,28^ To select the optimal pH value for the electrochemical sensing of NER, a pH range of 2.0–12.0 was investigated using different supporting electrolytes, including PBS, ABS, and BRB solutions. The oxidation peaks of NER (50 μM) in these supporting electrolytes were recorded at the BDDE using DPV. At the threshold pH values of 2.0 and 12.0, no peak was observed, which may be attributed to analyte degradation under strongly acidic and alkaline conditions. The observed peaks remaining pH values are illustrated in Figure 1. Oxidative peaks were obtained in all investigated electrolytes within the potential window extending from +0.30 to +1.00 V.

**Figure 1:**
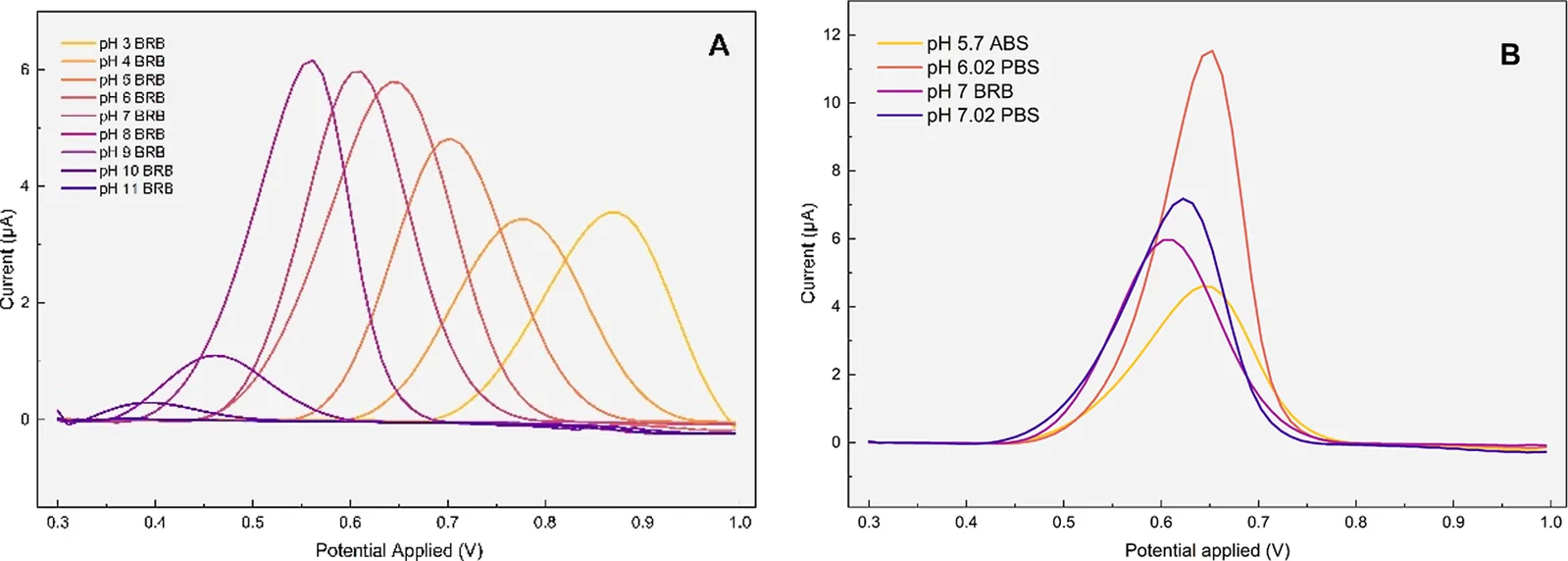
DPV responses of NER (50 μM) at the BDDE under different electrolyte and pH conditions: (A) BRB solutions over the pH range of 3.0–11.0; (B) comparison of selected electrolyte conditions: ABS (pH 5.7), PBS (pH 6.02), BRB (pH 7.0), and PBS (pH 7.02).

In addition, a pronounced shift in the NER peak potentials toward lower values was observed with increasing pH (Figure 1A,B), suggesting proton involvement in the electrode reaction.^29^ Also, this result indicates that the oxidation of NER occurs facilitated at these pH values.

An understandable pH-dependent displacement of the NER oxidation potential was noticed in BRB medium (Figure 2A). As the solution became less acidic, Ep gradually moved toward less positive potential (vs Ag/AgCl, 3 M KCl). Curiously, this dependency was not defined by a single linear trend but consisted of two distinct regions separated at approximately pH 9.0. The first department, extending from pH 3.0 to 9.0, indicated a pronounced potential shift with a slope of −66.6 mV/pH (eq 1), whereas above pH 9.0 the pH dependence became significantly weaker, giving a slope of only −2.2 mV/pH between pH 9.0 and 11.0 (eq 2). The changeover between these two regions happens close to the announced p*K*_a_ of NER (8.81, ChemAxon),^201^ suggesting that the alteration in the Ep–pH relationship is associated with a change in the protonation state of the molecule. This correspondence between the electrochemical breakpoint and the acid–base features of NER further support the contribution of proton-coupled processes to its oxidation at the BDDE. The two pH-dependent areas are represented by the following regression equations

1 $$E_{P}\;\left(mV\right)=-66.6pH+1054.6\;\left(pH3.0-9.0\right)\left(R^{2}=0.99\right)$$

2 $$E_{P}\;\left(mV\right)=-2.2pH+473.5\;\left(pH9.0-11.0\right)\left(R^{2}=0.75\right)$$

**Figure 2:**
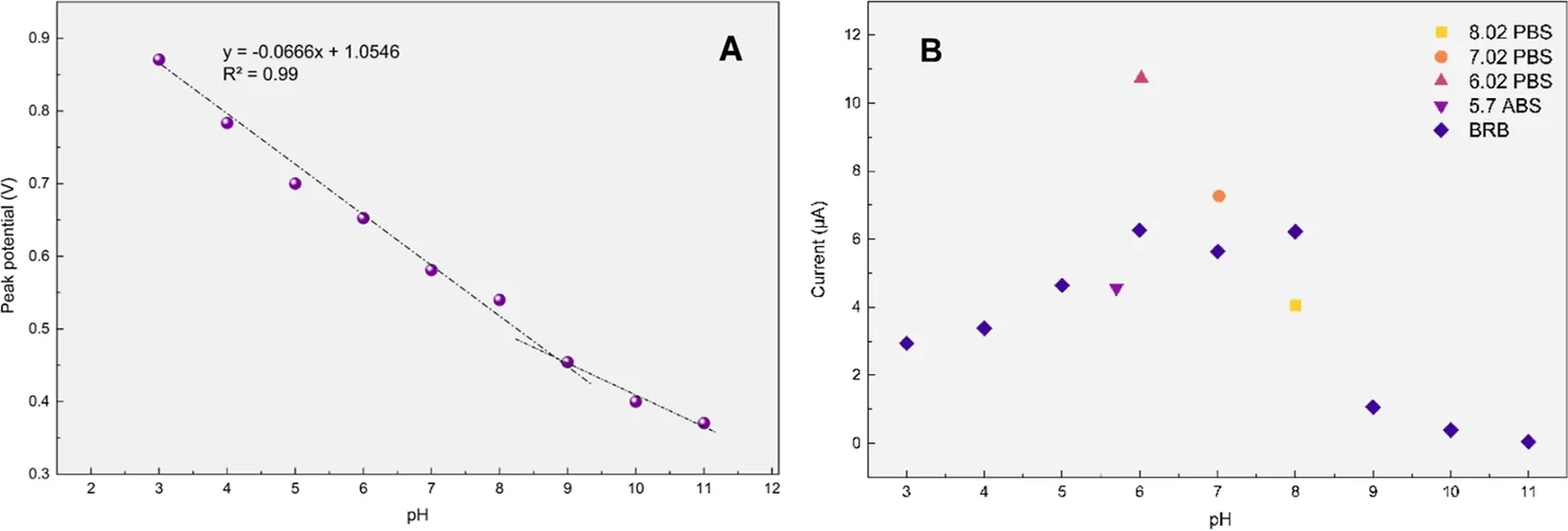
Variation of the peak potential of NER as a function of pH obtained from DPV voltammograms presented in Figure 1A (A) and influence of pH on the anodic peak current of NER at the BDDE in different supporting electrolytes obtained from DPV measurement (B).

One of the remarkable findings of this experiment is the establishment of a clearly expressed linear correlation between the oxidation peak potential of NER and solution pH, as described by the above regression equation. The satisfactory linearity of this relationship reflected a high regression coefficient (*R* ^2^ = 0.99) between pH 3–9, confirms the strong influence of pH on the anodic electrochemical behavior of NER. Furthermore, the slopes of approximately −66.6 mV/pH and −2.2 mV/pH obtained from this dependence provide mechanistic insights into the simultaneous involvement of electrons and protons in the irreversible oxidation of NER on the surface of BDDE. The slope of −66.6 mV pH^–1^ obtained in the pH 3.0–9.0 region approaches the theoretical Nernstian value of 59.2 mV pH^–1^, supporting an approximately 1:1 proton-to-electron stoichiometry in the oxidation process of NER.

A prediction of the electron-transfer number was acquired from the peak characteristics of DPV. For NER oxidation at the BDDE, the separation between the peak potential and the corresponding half peak potential, stated as Δ*E*_p_ = *E*_p_ – *E*_p_/_2_, was 83.8 mV. This experimentally derived Δ*E*_p_ was then utilized in eq 3 to compute the number of electrons transferred during the oxidation procedure.

3 $${\Delta E}_{p}{=E}_{p}-E_{p/2}=\left(\frac{47.7}{\alpha n}\right)mV$$

For a completely irreversible electrochemical reaction, taking α as 0.5 for an irreversible electrode process,^27^ application of eq 3 gave an *n*_a_ value of 1.14. Accordingly, the experimental data was rounded to unity, demonstrating that NER oxidation at the BDDE proceeds through a one-electron transfer step. Comparable irreversible electrochemical behavior has been documented in the literature.^30^ The effect of pH values on the electroanalytical response was orderly evaluated. Various buffer solutions were investigated for the most convenient electroanalytical performance in terms of sensitivity, repeatability, and an improved signal-to-baseline ratio. As observed in (Figure 2B), the peak current exhibited a strong dependence on pH, indicating that proton-coupled electron transfer plays a significant role in the oxidation process. This study, conducted to determine which type of buffer yielded the best results in terms of peak current height, peak sharpness, and reproducibility, showed that the highest peak currents were obtained between 6.02 and 8.02 pH values. NER response was monitored at the lowest pH 3.0. In BRB (pH 3.0–11.0), the peak current increased regularly by increasing pH up to pH 6.0, achieving a maximum value, and then diminished sharply after pH 8.0, which may be attributed to alterations in the protonation state of the NER and/or abated electrochemical activity under alkaline conditions.

Among the phosphate buffer systems, the highest peak current for NER was obtained at pH 6.02 PBS. Nevertheless, the enhanced signal intensity, the voltammetric response at this pH was found to be poorly reproducible, exhibiting significant peak instability and fluctuations in current magnitude between consecutive measurements. Such behavior indicates either an unstable solution–electrode interface or the presence of competing chemical equilibria affecting the electroactive species. In contrast, PBS at pH 7.02 exhibited a slightly lower peak current but showed outstanding repeatability, stability, and a well-defined peak shape with a stable baseline. Based on overall electroanalytical performance rather than peak height alone, 0.1 M PBS at pH 7.02 was selected as the optimal supporting electrolyte for subsequent analyses. This pH also provided stable and well-defined current responses with a distinct peak shape. At this pH, considering the reported p*K*_a_ value of NER (8.81), the molecule predominantly exists in a partially protonated form, which facilitates controlled electron transfer at the BDDE surface without significant adsorption or signal distortion.

### 3.2 Surfactant-Mediated Modulation of the NER Response

The electrochemical behavior of NER on the surface of BDDE was studied using DPV in various buffer solutions using with and without different surfactants. Surfactant molecules can rearrange the local environment of an electroactive species through molecular association and interfacial interplays, thereby making alterations to parameters such as current intensity, peak potential, and analytical sensitivity. The existence of surfactant molecules in the electrochemical medium provides the formation of micellar structures, which can encapsulate electroactive molecules and modify their local environments. Such micellization influence the electroanalytical response by changing the redox potential and, therefore, the detection sensitivity.^19^ In this study, the impact of different surfactants on the electro-oxidation of NER was evaluated using CTAB as a cationic surfactant, SDS as an anionic surfactant, and Tween 20 as a nonionic surfactant. The effect of surfactant concentration was investigated across concentrations spanning 10.0–500.0 μM, whereas 50 μM was employed for CTAB and SDS. Notably, the CMC value of Tween 20 (CMC = 60 μM) fell within the investigated concentration range, allowing its electrochemical reply to be investigated both below and above the CMC.

Alterations in peak potential, peak current, peak shape, and repeatability were analyzed to elucidate surfactant-analyte-electrode interactions. The results obtained are shown in Figure S2. In the absence of surfactants, NER (5 × 10^–5^ M) exhibited a well-defined and reproducible anodic peak with a low relative standard deviation (RSD ≈1.30%) at pH 7.02 PBS. Upon addition of CTAB (50 μM), a noticeable shift of the anodic peak toward more positive potential was noticed, accompanied by a moderate decrease in peak current compared to the surfactant-free system. The increased RSD value (≈2.65%) indicates a deterioration in signal repeatability. This behavior can be attributed to electrostatic repulsion between the positively charged headgroups of CTAB and the partially protonated NER molecules at pH 7.02. In addition, adsorption of CTAB onto the BDDE surface likely forms a positively charged interfacial layer which hinders the efficient approach of NER to the electrode and adversely affects mass transport and electron transfer kinetics. In contrast, the existence of the anionic surfactant SDS (50.0 μM) resulted in a pronounced decrease in peak current, although the signal remained highly reproducible, as reflected by a low RSD value (≈1.29%). The negatively charged sulfate head groups of SDS can electrostatically interact with the protonated sites of NER, facilitating partial incorporation of the analyte into interfacial structures. This solubilization effectively decreases the concentration of freely diffusing electroactive NER species available at the surface of BDDE and leads to suppressed current responses despite satisfactory repeatability. It should be noted that the concentrations of SDS and CTAB used in this study (50 μM) are well below their reported CMC (approximately 8.0–9.0 mM for SDS and 0.8–1.0 mM for CTAB). Therefore, the observed electrochemical effects are likely associated with surfactant monomers and their interactions at the electrode–solution interface rather than micelle formation.

The effect of the nonionic surfactant Tween 20 was strongly concentration dependent. At lower surfactant concentrations (10.0 and 50.0 μM), Tween 20 produced relatively high peak currents; however, these responses were accompanied by poor repeatability, as evidenced by elevated RSD values (≈3.29–3.40%). The absence of electrostatic interactions in nonionic surfactants suggests that the observed signal instability results from dynamic adsorption–desorption processes and interfacial organization, since it modifies the local interfacial microenvironment at the electrode surface. Increasing the concentration of Tween 20 to 100 μM produced a pronounced amplification in the anodic current of NER, which may be associated with improved incorporation and solubilization of the target analyte within Tween 20 micellar assemblies. However, further increases in Tween 20 concentration (500 μM) resulted in a decline in electroanalytical performance and increased signal variability, presumably due to excessive micellar coverage at the electrode–solution interface, which hinders electron transfer and alters diffusion pathways. Based on the surfactant optimization researches, further experimental studies were performed in the presence of 100 μM Tween 20 (which is above its reported CMC of 60 μM). Accordingly, all calibration measurements and real sample analyses were carried out at 100 μM Tween 20, as this condition provided a sufficient signal response while maintaining acceptable repeatability.

Tween 20 adsorbs at the BDDE/electrolyte interface and modifies the interfacial properties by forming a relatively loose and dynamic layer. In this configuration, the hydrophobic regions of Tween 20 interact selectively with hydrophobic domains on the electrode surface, while the hydrophilic polyoxyethylene chains extend into the aqueous phase. This interfacial organization creates a favorable microenvironment that supports the local accumulation and stabilization of NER molecules near the electrode surface. As a result, both mass transport and interfacial electron transfer processes are facilitated, leading to a significant increase in the voltammetric peak current.

Importantly, the intensification observed with Tween 20 should not necessarily be interpreted as a common acceleration of electron transfer at the BDDE. Rather, the outcomes point to a more complicated contribution from surfactant-induced alterations in the local interfacial environment and probable NER–surfactant interplays. To further distinguish between these possibilities and examine how Tween 20 changes the electrolyte/BDDE interface, EIS was subsequently employed.

### 3.3 Interfacial Characterization by Electrochemical Impedance Spectroscopy

To further clarify whether the existence of Tween 20 alters the interfacial characteristics of the BDDE, EIS measurements were performed employing the ferri/ferrocyanide redox couple as an electrochemical probe. A substantial enlargement of the Nyquist semicircle was perceived after the adding of Tween 20, reflecting a pronounced promotion in the interfacial charge transfer resistance (*R*_ct_). Quantitatively, *R*_ct_ altered from 510 Ω for the surfactant-free buffer to 1822 Ω in the Tween 20-containing medium, representing an increase of more than three times. This pronounced increase suggests that Tween 20 modifies the electrode–electrolyte interface and hinders the electron transfer of the ferri/ferrocyanide redox probe, possibly through the formation of a partially blocking interfacial layer.

Furthermore, in the existence of Tween 20, the impedance signal deviates from the ideal semicircular shape which indicates the emergence of additional interfacial processes and a more heterogeneous interface of electrode/electrolyte. Such deviation may reflect alterations in interfacial organization and diffusion-related contributions induced by Tween 20. This deviation proposes a more heterogeneous interfacial environment, potentially involving additional procedures that may also affect diffusion behavior.

To eliminate the possible contribution of differences in solution conductivity, the Tween 20 solution was prepared in the same supporting electrolyte (pH 7.02 PBS), thereby ensuring a constant ionic strength across all measurements. The ferri/ferrocyanide redox couple provides a reliable and sensitive probe for evaluating interfacial properties due to its well-defined and reversible electron-transfer behavior. Therefore, the observed increase in *R*_ct_ maintains additional argument that Tween 20 reorganizes the BDDE/electrolyte interface and changes the accessibility of the redox probe to the electrode surface.

It should be noted that the increase in charge-transfer resistance observed in the EIS measurements does not necessarily contradict the enhancement of the NER oxidation signal observed in DPV. The EIS experiments were performed using the ferri/ferrocyanide redox couple, and the increased *R*_ct_ value indicates that Tween 20 modifies the electrode–electrolyte interface and influences the electron-transfer kinetics of the probe species. In contrast, the voltammetric response of NER reflects the oxidation behavior of the analyte itself. Therefore, the enhanced NER peak current despite the increased *R*_ct_ may be attributed to favorable analyte–surfactant interactions and changes in the interfacial microenvironment induced by Tween 20, which may favor the local availability of NER for electrooxidation at the BDDE.

### 3.4 Electrochemical Kinetics and Mass-Transport Characteristics of NER

Scan rate dependent CV measurements were engaged to acquire further understanding into the oxidation characteristics and mass-transport behavior of NER at the BDDE.^29^ For this reason, the anodic reply of NER was observed at scan rates ranging from 10 to 1000 mV s^–1^, and the outcoming variations in peak current and peak potential were assessed. These experimental studies also enabled assessment of whether diffusion or surface adsorption predominantly governs the electrode process. All measures were carried out in the previously chosen PBS medium at pH 7.02. The blank electrolyte produced no detectable faradaic signal within the investigated potential window. Following the addition of 0.50 μM NER, however, a distinct anodic signal emerged at approximately +0.81 V vs Ag/AgCl (Figure S3), which was assigned to oxidation of NER. The nonexistence of a corresponding reduction peak during the reverse potential scan demonstrates that the electrochemical oxidation of NER at the BDDE is irreversible.

The dependency of peak current on scan rate supplies a functional criterion for identifying the dominant mass-transport system of an electrode process. A slope approaching 0.5 in the log Ip–log *v* relationship is usually associated with diffusion control, whereas a merit near 1.0 is characteristic of an adsorption-dominated answer. For NER, linear regression yielded with a slope of 0.5898 (Figure 3A, eq 4), which lies greatly closer to the theoretical diffusion-controlled value.^30^ This outcome recommends that the anodic response is directed predominantly by diffusion of NER from the bulk solution toward the BDDE interface, with surface adsorption making a comparatively minor contribution.^31^

4 $$\log Ip\;\left(\mu A\right)=0.5898\log v\;\left(mV{\;s}^{-1}\right)-1.7194\;with{\;R}^{2}=0.9938$$

**Figure 3:**
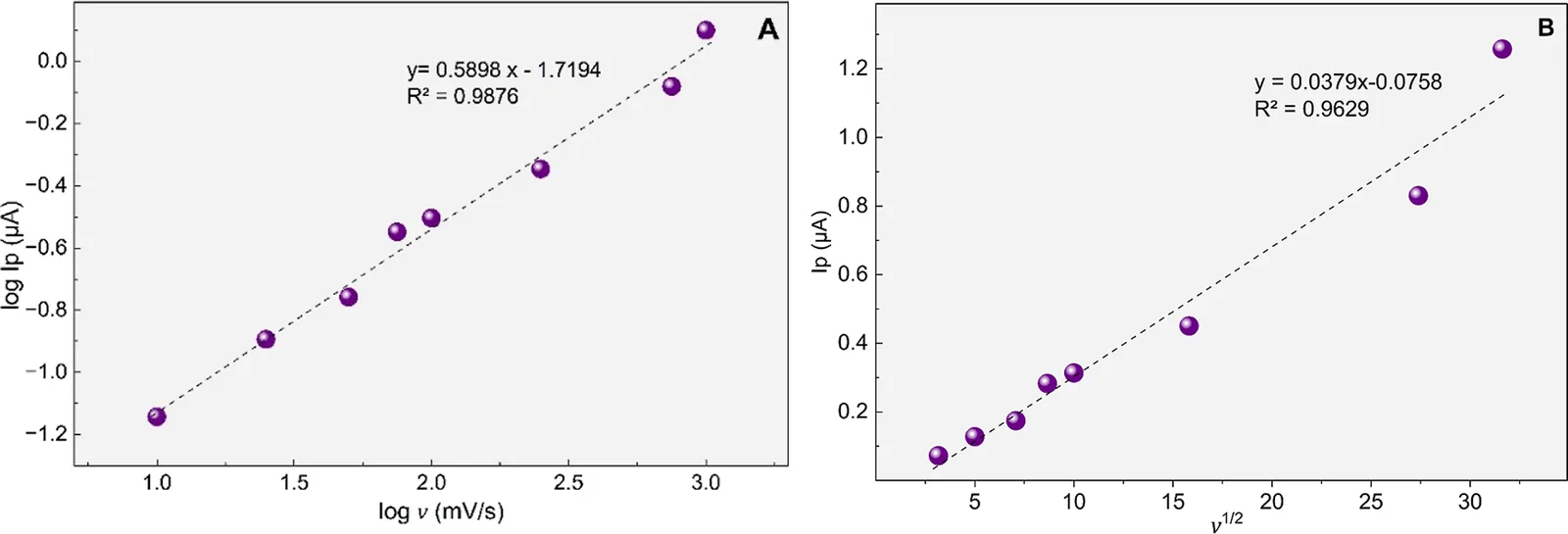
Log Ip vs log *v* (A), Ip vs *v* ^1/2^ (B) graphs of 5.0 × 10^–7^ M NER in pH 7.02 PBS obtained in the range of 10–1000 mV s^–1^.

Further support for diffusion-dominated NER oxidation was ensured by the linear dependency of the anodic peak current on the square root of scan rate (*v* ^1/2^), as described by eq 5 (Figure 3B).^32^

5 $$Ip\;\left(\mu A\right)=0.038\;v^{1/2}{\left(mV{\;s}^{-1}\right)}^{1/2}-0.076\;with{\;R}^{2}=0.9813$$

Overall, the experimental results show that oxidation of NER at the BDDE is predominantly governed by diffusion rather than surface adsorption, in agreement with the relatively low adsorption tendency generally reported for BDDE surfaces.^33,34^

### 3.5 Proposed Electrooxidation Mechanism of NER

The CV answer of NER at the BDDE was described by a single anodic signal without a corresponding reduction peak during the opposite potential scan (Figure S3), exhibiting the irreversible nature of the electrode process. The incident of only one oxidation signal remarks that the voltammetric reply is predominantly related to a single electroactive site within the NER molecule. Mechanistic knowledge was further had from the pH-dependent potential shift and the assay of the irreversible peak parameters. The progressive displacement of the anodic potential toward less positive values with increasing pH, together with a slope approaching the theoretical Nernstian value, promotes proton participation in the oxidation step. Moreover, evaluation of Δ*E*_p_ = *E*_p_ – *E*_p/2_, assuming an electron-transfer coefficient (α) of 0.5, delivered an electron number close to unity (*n* ≈ 1). Consequently, the electrochemical verification is consistent with a proton-coupled oxidation pathway involving approximately one electron and one proton.

To identify the reactive site involved in the electrode process involved in the oxidation process, the electrochemical behavior of NER was compared with the well-established electrooxidation mechanism of alkoxy/methoxy-substituted aromatic ethers. In a related voltammetric framework, Tamsulosin, Formoterol, Mebeverine HCl, Sulpiride, Verapamil, and the model compounds Anisole and 4-Methoxyphenol have been investigated as representative systems for oxidation of the methoxybenzene moiety, and the consistency among these responses has been used to rationalize drug determination via alkoxybenzene oxidation. In addition, the electrochemical behavior and oxidation mechanism of Formoterol Fumarate was discussed by Demircigil et al., providing a precedent for mechanistic interpretation of pharmaceuticals containing electron-rich aryl-ether environments. The aromatic framework of NER bears electron-donating aryl ether and alkoxy moieties, with the inclusion of pyridinylmethoxy and ethoxy substituents. These chemical groups make richer the π-system in electron density, thereby favoring anodic electron removal and the subsequent creation of a radical-cation intermediate. Radical-cation formation is a widely recognized first step in the anodic oxidation of substituted methoxybenzenes and dialkoxybenzenes.^31–200^ Based on the electrochemical behavior of NER, the Ep–pH relationship, the estimated electron-transfer number, and literature reports describing the oxidation of alkoxy/methoxy-substituted aromatic compounds, a tentative oxidation pathway involving the alkoxybenzene moiety is proposed in Figure 4. This proposed mechanism should be regarded as an interpretation rather than a definitive proof of the oxidation site, as no direct identification of oxidation products was performed. Nevertheless, the hypothesis is consistent with previous electroanalytical studies in which the oxidation of pharmaceuticals containing alkoxy/methoxy-substituted aromatic groups was interpreted by analogy with the redox behavior of simple aryl ether model compounds such as anisole and 4-methoxyphenol.

**Figure 4:**
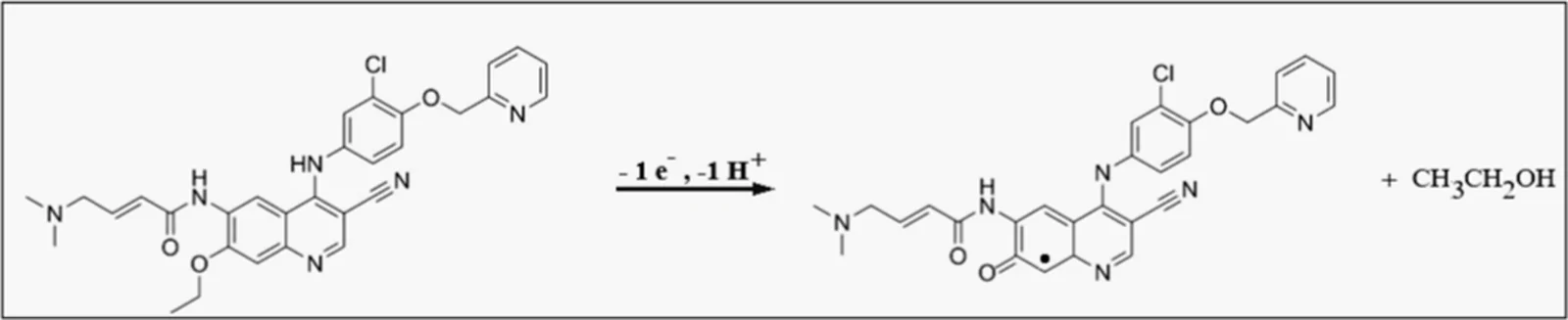
Schematic representation of the proposed NER oxidation pathway at the BDDE. ^+^.

Taken together, the irreversible voltammetric attitude, pH-dependent potential shift, and guessed one-electron transfer promote a proton-coupled oxidation pathway for NER. When taken into account alongside the electronic characteristics of the suggested oxidation site, these electrochemical results provide the experimental basis for the oxidation pathway illustrated in Figure 4.

### 3.6 Computational Support for the Proposed Oxidation Pathway

The electronic characteristics of NER were further explored computationally utilizing the semiempirical PM6 Hamiltonian at the singlet state. Following ground-state geometry optimization, the total energy and Frontier molecular orbital energies were computed together with the corresponding chemical descriptors. Particular awareness was also shown to the spatial localization of the highest occupied molecular orbital (HOMO) and lowest unoccupied molecular orbital (LUMO), ensuring knowledge on the electronic distribution within the NER structure. The resulting optimized geometry, including the relevant structural features and atom labeling, is illustrated in Figure 5.

**Figure 5:**
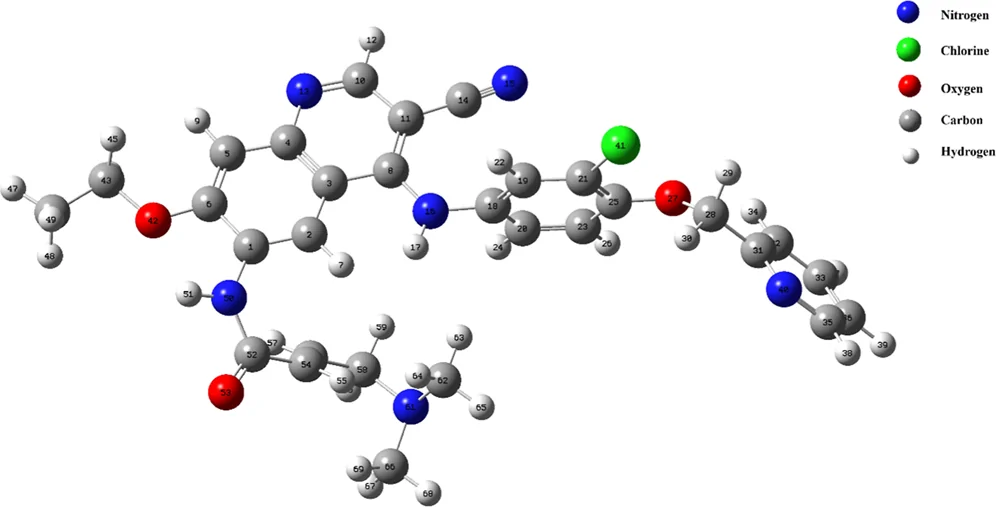
Optimized and labeled structures of the NER via semiempirical calculation using the PM6 Hamiltonian.

Frontier molecular orbital analysis was employed to feature the electronic distribution of NER and to specify molecular regions potentially involved in its reactivity. The HOMO map submitted in Figure 6 demonstrates pronounced orbital density across the conjugated aromatic system and around heteroatom-bearing moieties, proffering that these regions are preferentially involved in electron-donating behavior during the electrochemical oxidation of NER. The LUMO is located on adjacent π-conjugated domains, suggesting the possibility of charge redistribution following electron transfer. The calculated energy values (*E*_LUMO_ = −0.03939 au and *E*_HOMO_ = −0.32812 au) correspond to an energy gap (Δ*E*_Gap_ = 0.28873 a.u, corresponding to 7.86 eV), which reflects a relatively small separation between Frontier orbitals and may be associated with a NER molecule that can participate in electron transfer processes. So, these values obtained from theoretical studies can serve as supportive evidence for interpreting the electrochemical behavior of NER. The electronic distribution depicted in Figure 4 highlights conjugated and heteroatom-bearing moieties as electron-rich regions, suggesting their possible contribution to the electron-transfer step involved in NER oxidation.

**Figure 6:**
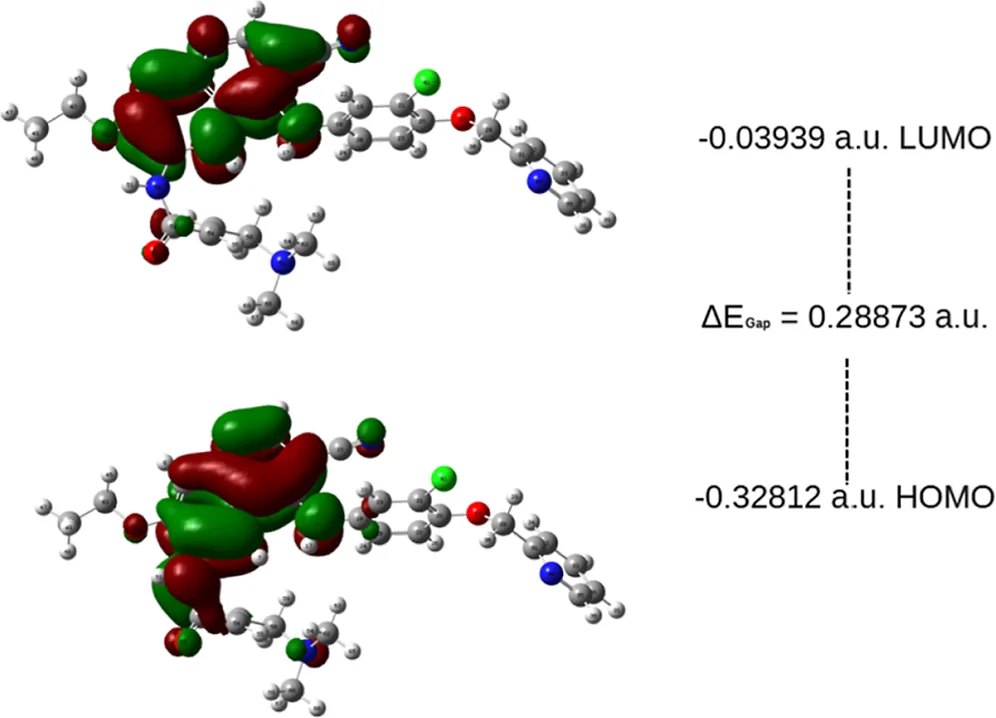
HOMO and LUMO distributions, corresponding orbital energies, and calculated energy gap (Δ*E*_gap_) of NER.

### 3.7 Analytical Performance of the Proposed Voltammetric Method

The quantitative answer of the designed method was installed under the optimal experimental conditions by correlating the anodic peak current with NER concentration. Figure 7 represents the DPV profiles obtained in 0.1 M PBS (pH 7.02) containing 100 μM Tween 20 after successive additions of NER. Increasing the target analyte concentration from 0.03 to 1.50 μM resulted in a progressive increment in the oxidation current. Within this concentration interval, the current reply demonstrated linear dependence on NER concentration, as shown in the inset of Figure 7. Linear regression of the experimental data produced the following calibration equation: *y* (μA) = 0.3243­(NER, μM) – 0.0107, with *R* ^2^ = 0.9931. In this way, a linear analytical range of 0.03–1.50 μM was obtained for NER determination using the BDDE/Tween 20 system.

**Figure 7:**
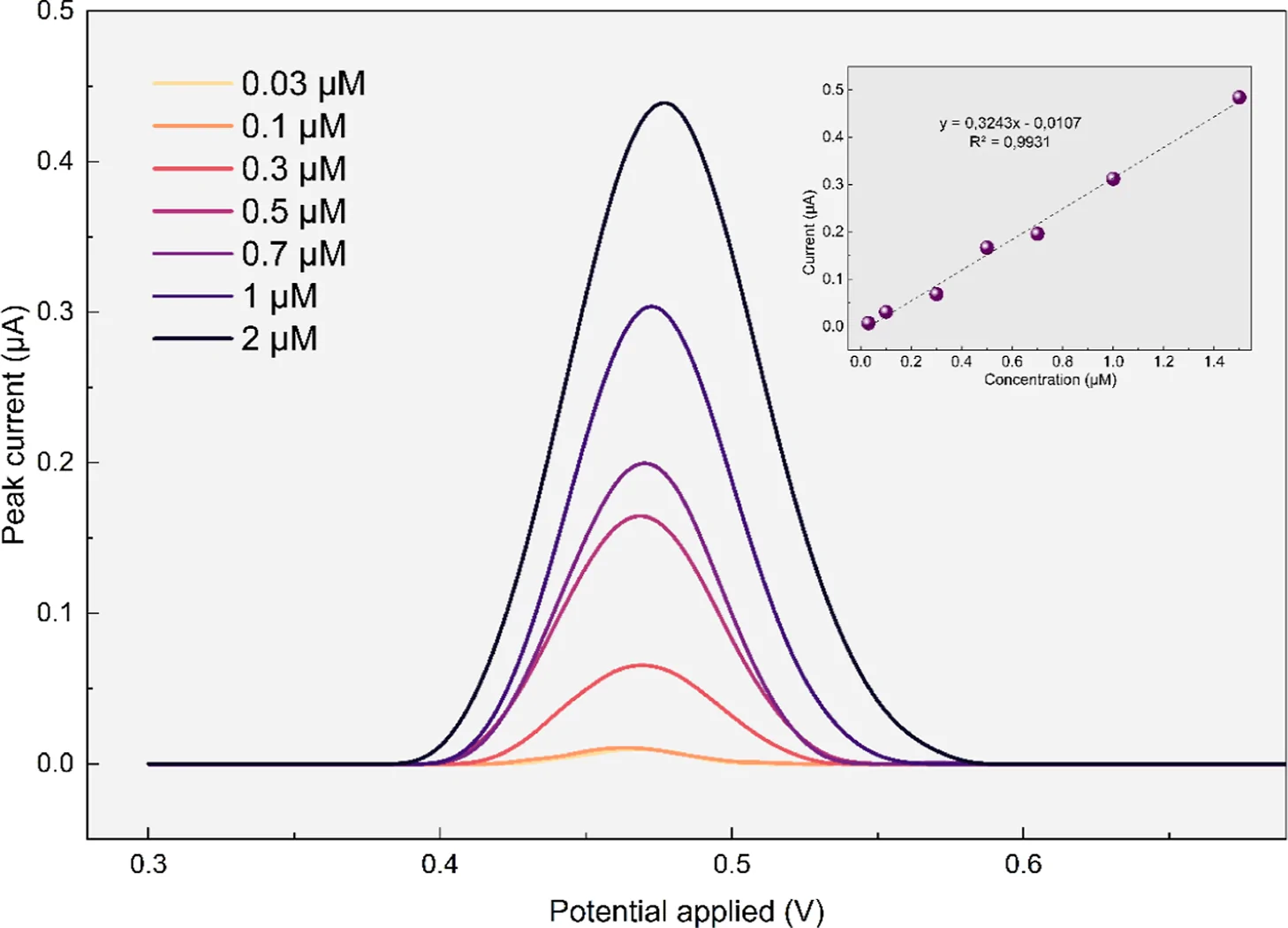
DPVs of increasing concentrations of NER at BDDE in 0.1 M PBS at pH 7.02 with 100 μM Tween 20. (inset) A graph of peak response versus NER concentration.

The analytical detection and quantification limits (LOD and LOQ) were determined using signal-to-noise (S/N) criteria of 3 and 10, respectively, in accordance with the established approach. The corresponding LOD and LOQ values were calculated using eqs 6 and 7

6 $$LOD=\frac{3S_{b}}{m}$$

7 $$LOQ=\frac{10S_{b}}{m}$$

In eqs 6 and 7, *S*_b_ denotes the standard deviation of the blank or baseline signal, while m corresponds to the slope of the calibration curve and symbolizes the analytical sensitivity. The numerical factors 3 and 10 were applied for prediction of the LOD and LOQ, respectively. Based on these parameters, an LOD of 5.09 nM, corresponding to 2.84 ng mL^–1^, was obtained for NER (Table 1). This low LOD demonstrates the ability of the method to quantify NER at trace concentration levels, a prominent consideration for analysis in complicated matrices. The corresponding LOQ was determined as 15.16 nM (8.44 ng mL^–1^). In addition, the calibration slope yielded a sensitivity of 0.3243 μA μM^–1^. Collectively, the wide linear response and low detection and quantification limits demonstrate the favorable analytical performance of the proposed voltammetric approach for NER determination.

**Table 1:** Analytical Figures of Merit for NER in Standard Solution and Serum in pH 7.02 PBS and 100 μM Tween 20 Using DPV

	standard	serum
measured potential (mV)	471	471
linear range (μM)	0.03–1.50	0.05–1.50
slope (μA/μM)	0.3243	0.4124
intercept (μA)	–0.0107	–0.0174
the coefficient of determination (*R* ^2^)	0.9931	0.9966
LOD (μM)	0.00509	0.00227
LOQ (μM)	0.01516	0.00687
intraday precision of peak current (RSD %)^a^	1.24	-
interday precision of peak current (RSD %)^a^	3.18	-

a Based on five replicate measurements. RSD: relative standard deviation.

Although numerous methods for NER determination have been reported in the literature, most of these approaches rely on chromatographic techniques. A comparison of the proposed DPV method with previously reported analytical methods for NER determination is summarized in Table 2.

**Table 2:** Comparison of Reported Analytical Approaches for the Determination of NER^a^

method	linear range	LOD	application	references
UPLC-MS/MS	0.5–500 ng mL^–1^	0.5 ng mL^–1^	human plasma samples	^10^
UPLC-MS/MS	10.0–5000.0 ng mL^–1^	-	rat plasma samples	^8^
UPLC-MS/MS	4.0–500.0 ng mL^–1^	4.0 ng mL^–1^	human plasma samples	^11^
spectrofluorimetric	0.1–10.0 μg mL^–1^	0.07 μg mL^–1^	tablet samples	^9^
HPLC	7–42 μg mL^–1^	0.4480 μg mL^–1^	pharmaceutical formulations	^36^
DPV	16.70–836.00 ng mL^–1^	2.84 ng mL^–1^	commercial human serum samples	this study

a DPV: Differential Pulse Voltammetry, LOD: Limit of Detection, MS: Mass Spectrometry, UPLC: Ultra Performance Liquid Chromatography.

UPLC-MS/MS methods provide wide linearity ranges and high sensitivity; however, they require labor-intensive sample preparation, expensive instrumentation, and trained personnel, which may limit routine applicability in clinical studies. In contrast, spectrofluorimetric methods offer operational simplicity but suffer from relatively narrow linearity ranges and lower sensitivity, particularly when applied to complex matrices.

The developed DPV method exhibits a competitive linear range (16.70–836.00 ng mL^–1^) and a low detection limit (2.84 ng mL^–1^), which is comparable to or better than several reported methods, while enabling direct analysis of commercial human serum samples. The high sensitivity and low LOD achieved by the proposed electrochemical method demonstrate its potential as a feasible and price-effective option to traditional chromatographic methods.

As shown in Table 2, the proposed DPV method exhibits a markedly LOD than the reported HPLC method. While the HPLC procedure provided an LOD of 0.4480 μg mL^–1^ (i.e., 448.0 ng mL^–1^),^36^ the developed voltammetric method achieved an LOD of 2.84 ng mL^–1^, corresponding to an approximately 158-fold improvement in detection capability. Moreover, the obtained LOD is also lower than that reported for one of the published UPLC-MS/MS methods (4.0 ng mL^–1^).^11^ These results highlight that the proposed electrochemical approach is not only simpler and more cost-effective, but also highly sensitive for the trace-level determination of NER.

In addition, the developed electrochemical method offers significant advantages in terms of cost-effectiveness, simpleness, time-efficient analysis, and least sample pretreatment. These properties, together with its satisfactory electrochemical performance, highlight the potential of the developed DPV method as a practical and reliable alternative for routine determination of NER in biological samples.

### 3.8 Analytical Evaluation of NER Determination in Human Serum

The analytical reply obtained in uncomplicated supporting electrolytes may vary from that noted in biological samples because of the existence of endogenous serum constituents. Consequently, the quantitative performance of the suggested technique was additionally investigated directly in human serum under the optimized experimental conditions. An independent matrix-based calibration was constructed to investigate the linear response, sensitivity, and detection capability of the method in the presence of serum components, thereby assessing its suitability for NER determination in real biological samples.

A linear calibration relationship for NER in serum was obtained over the concentration range of 0.05–1.50 μM (Figure S5), demonstrating the suitability of the method for quantitative analysis in complex biological media. The calibration plot exhibited a slope of 0.4124 μA μM^–1^ and an intercept of −0.0174 μA, with an excellent correlation coefficient (*R* ^2^ = 0.9966), confirming the reliability of the calibration model in the serum matrix (Figure S5 **inset)**.

The LOD and LOQ values were calculated as 2.27 nM and 6.87 nM, respectively indicating that the proposed method is capable of detecting NER at trace levels in serum samples.

The practical applicability of the proposed BDDE-based procedure was examined in commercially available human serum. NER quantification in this matrix was carried out by the standard addition approach, allowing the accuracy of the voltammetric determination to be evaluated in the presence of serum constituents. For this purpose, serum aliquots were fortified with a known NER concentration of 0.2 μM and analyzed by DPV using the previously optimized experimental parameters. Five replicate determinations (n = 5) were performed, and the resulting data were used to assess recovery and measurement reproducibility. The BDDE exhibited high recovery efficiency as 99.98 ± 2.79% for NER in commercial human serum samples, confirming the accuracy and practical applicability of the proposed sensing method.

### 3.9 Interference Studies

Selectivity is an essential parameter for evaluating the sensing performance of an electrode in practical applications. Since one of the aims of the present work is the analysis of NER in real samples such as human serum, potential interfering species, including NaCl, KCl, caffeine, glucose, and ascorbic acid, were selected. Voltammetric measurements for all interfering substances were performed in triplicate, and the average responses were used to Figure S6.

The BDDE sensor was evaluated in the presence of interfering species at concentration ratios of 1:1, 1:10, and 1:100 relative to 0.5 μM NER. Figure S6 presents sensor responses to different interferents compared with the response of NER alone. The sensor demonstrated RSD values ranging from 0.99% to 5.72%, with recovery values between 97.01% to 108.83% in the presence of interfering substances, indicating satisfactory analytical performance. It was observed that the voltammetric signal of 0.5 μM NER was not significantly affected by equal concentration of NaCl, KCl, caffein, ascorbic acid, and glucose. Evaluation of the results (Table 3) further confirmed that none of the tested species produced a significant interference effect on NER determination at the BDDE, even at a 1:100 ratio. These findings indicate that the BDDE exhibits high selectivity and is not significantly influenced by common interfering species, making it suitable for accurate NER determination in real biological samples.

**Table 3:** Effect of Some Interference Species towards the Response of NER at BDDE Sensor (*n* = 3)

interferent	recovery %		
(*C*_Int_/*C*_NER_)	1:1	1:10	1:100
NaCl	97.91 ± 2.17	101.39 ± 3.87	103.06 ± 2.12
KCl	100.14 ± 1.70	103.22 ± 1.30	108.83 ± 5.72
caffein	97.01 ± 3.53	103.52 ± 2.77	104.17 ± 3.27
ascorbic acid	102.64 ± 3.93	102.57 ± 1.79	105.14 ± 0.99
glucose	98.33 ± 1.38	97.44 ± 3.05	105.92 ± 3.22

### 3.10 Greenness Assessment of the Proposed Electrochemical Method

The environmental sustainability of the proposed electrochemical method was assessed using three green analytical chemistry evaluation tools: AGREE, AGREEprep, and ComplexMoGAPI. These metrics evaluate different aspects of analytical greenness, including adherence to the twelve principles of Green Analytical Chemistry, the environmental impact of sample preparation, and the overall ecologic sustainability.

A score of 0.60 was obtained from the AGREE assessment. Indicating compliance with the principles of Green Analytical Chemistry (Figure 8). The method demonstrated several environmentally favorable characteristics, such as low energy consumption, the absence of derivatization, the use of a rapid electrochemical detection technique, and relatively low sample and reagent consumption. However, the overall score was affected by the offline analytical configuration, the necessity of serum sample preparation, and the use of acetonitrile during protein precipitation.^37^

**Figure 8:**
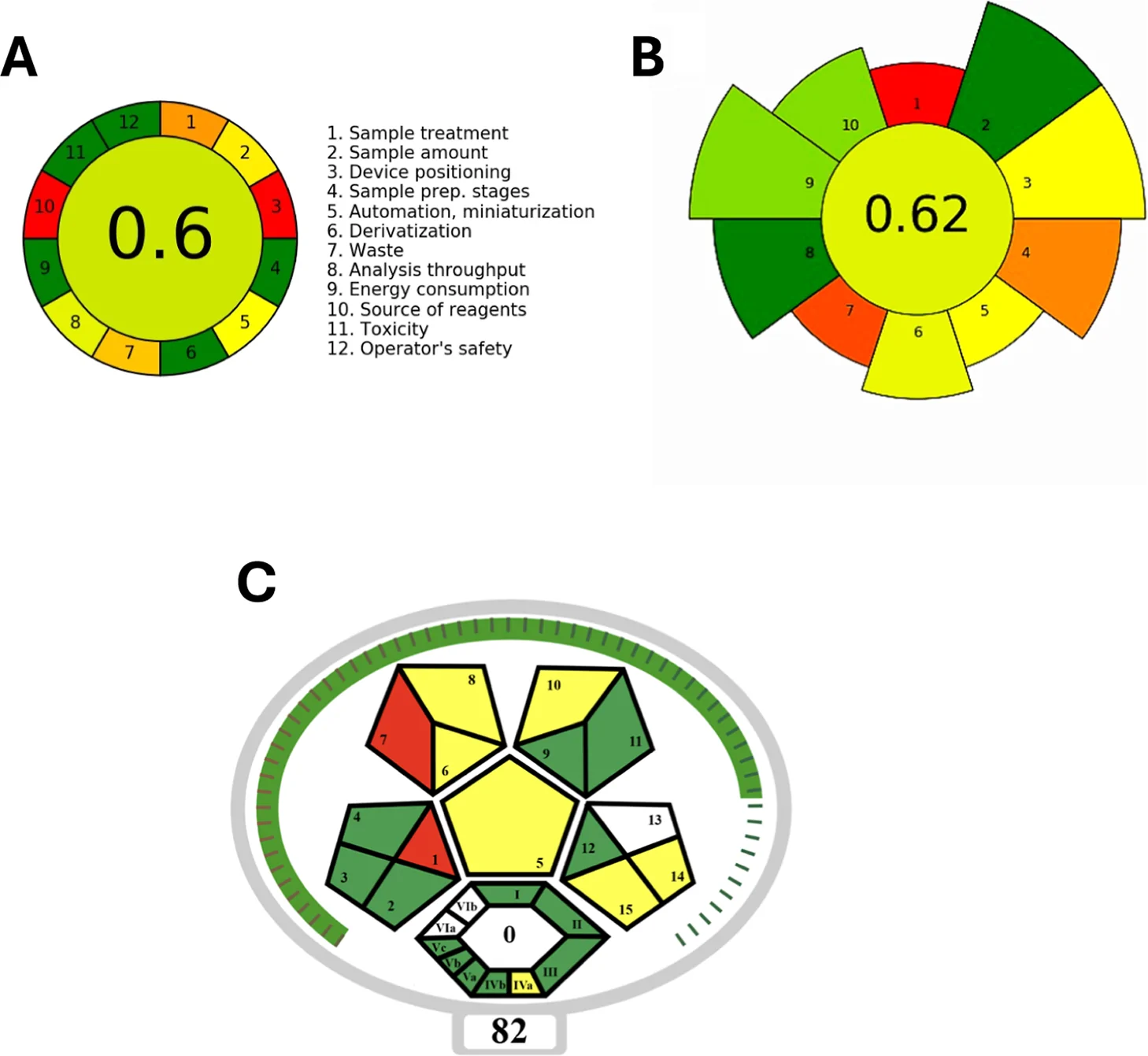
Greenness assessment of the proposed method using (A) AGREE (score = 0.60), (B) AGREEprep (score = 0.62), and (C) ComplexMoGAPI (score = 81/100). The three complementary metrics indicate that the proposed electrochemical method possesses good overall environmental sustainability while identifying the use of acetonitrile and offline sample preparation as the main factors limiting its greenness.

The AGREEprep evaluation resulted in a score of 0.62 (Figure 8), presenting a satisfactory level of sustainability for the sample preparation procedure. Although protein precipitation with acetonitrile was required before electrochemical analysis, the protocol involves only simple pretreatment step and requires small sample volume. The method generates a limited amount of waste and does not require derivatization or energy-intensive operations.^38^

For the further evaluations of the overall environmental profile, the proposed procedure was assessed using ComplexMoGAPI, which provided a satisfactory total score of 82/100 (Figure 8). The obtained score indicates that the method possesses good overall greenness despite the unavoidable use of a hazardous organic solvent during sample preparation. The favorable score comes from the absence of sample transportation and storage requirements, low energy consumption. Simple analytical workflow, minimal waste generation, and the use of an electrochemical technique requiring relatively simple instrumentation are the other advantageous criteria. The principal factors limiting the overall sustainability were associated with the use of acetonitrile and the offline sample preparation procedure.^39^

Overall, the results from these three greenness assessment tools demonstrate that the proposed voltammetric method provides a favorable environmental profile while maintaining excellent analytical performance. The complementary use of AGREE, AGREEprep, and ComplexMoGAPI offers an extensive evaluation of the method and confirms its suitability as a sustainable analytical approach for the determination of NER in pharmaceutical formulations and human serum samples.

## 4 Conclusion

This research presents, to the best of our information, the first systematic investigation of the electrochemical behavior of NER at an unmodified BDDE, together with the first evaluation of surfactant-mediated influences on its voltammetric reply. The electrochemical results proved that NER undergoes an irreversible and predominantly diffusion-controlled oxidation process involving approximately one electron and one proton. The pH-dependent behavior, scan-rate studies, and computational analysis collectively supported the suggested proton-coupled electrooxidation pathway, while the theoretical outcomes ensured complementary knowledge regarding the electron-rich molecular regions potentially involved in the oxidation process.

Among the examined surfactant molecules, the nonionic surfactant Tween 20 assembled the most favorable effect on the NER response, with 100 μM selected as the optimal concentration. Importantly, EIS measurements exhibited that the addition of Tween 20 increased the charge-transfer resistance of the ferri/ferrocyanide probe from 510 to 1822 Ω, despite the enhancement noticed for the oxidation current of NER. This evidently contrasting behavior designates that Tween 20 does not simply accelerate electron transfer at the BDDE. Instead, the findings support an analyte-dependent interfacial impact in which surfactant-induced organization of the electrolyte/BDDE microenvironment favors the electrochemical response of NER.

Under the optimized parameters of 0.1 M PBS at pH 7.02 containing 100 μM Tween 20, the suggested DPV technique provided a linear reply over 0.03–1.50 μM, with an LOD of 5.09 nM and an LOQ of 15.16 nM. The applicability of the method to a complicated biological matrix was further showed operating commercial human serum, where a linear range of 0.05–1.50 μM and a recovery of 99.98 ± 2.79% were procured. Moreover, common potentially interfering species produced no substantial effect on NER determination, supporting the selectivity of the developed procedure.

A further benefit of the suggested strategy is that sensitive NER determination is achieved without persistent modification of the BDDE, nanomaterial coatings, or biological recognition elements. The usage of an unmodified electrode together with surfactant-mediated interfacial enhancement decreases electrode preparation requirements and contributes to a effortless analytical workflow. In addition, the complementary greenness assessments using AGREE, AGREEprep, and ComplexMoGAPI indicated a favorable environmental profile for the method, while also identifying solvent use and offline sample preparation as the principal limitations to its sustainability.

Overall, the results establish the unmodified BDDE/Tween 20 system as a selective, sensitive, and comparatively uncomplicated platform for NER determination, while ensuring new mechanistic insight into how surfactant-mediated interfacial organization can direct the voltammetric response of NER. The combination of analytical performance, practicality to human serum, and favorable environmental characteristics focuses on the potential of this approach for further electroanalytical applications involving NER and structurally related pharmaceutical compounds.

## Supplementary Material



## Notes

It includes the chemical structure of NER (Figure S1), comparison of the anodic peak currents obtained in the absence and presence of different surfactants (Figure S2), cyclic voltammograms recorded at different scan rates (Figure S3), electrochemical impedance spectroscopy characterization of the bare BDDE in PBS buffer and Tween 20 solution (Figure S4), differential pulse voltammograms and the calibration plot for NER determination in commercial human serum samples (Figure S5), and interference studies demonstrating the effect of selected coexisting species on the analytical response (Figure S6) (PDF)

## Abbreviations

BDDE: boron-doped diamond electrode
BRB: britton–robinson buffer
CMC: critical micelle concentration
CTAB: cetyltrimethylammonium bromide
CV: cyclic voltammetry
DMSO: dimethyl sulfoxide
DPV: differential pulse voltammetry
EIS: electrochemical impedance spectroscopy
HER: human epidermal growth factor receptor
HOMO: highest occupied molecular orbital
LOD: limit of detection
LOQ: limit of quantification
LUMO: lowest unoccupied molecular orbital
NER: neratinib
PBS: phosphate-buffered saline
SDS: sodium dodecyl sulfate
WHO: world health organization
