# Supplementary material for: Electrochemical Behavior of Neratinib at a Boron-Doped Diamond Electrode: Role of Tween 20 in Interfacial Signal Enhancement
Source: ACS Omega. 2026 Sep 1;11(36):54221–33. doi: 10.1021/acsomega.6c05232 (PMC13584560; doi:10.1021/acsomega.6c05232)
Supplement: Supplementary file 1 [file ao6c05232_si_001.pdf]

Electrochemical Behaviour of Neratinib at a Boron-Doped Diamond Electrode: Role of Tween 20 in Interfacial Signal Enhancement

-SUPPLEMENTAL MATERIALS-

*Gulsum E. Meraki[1,2], Hulya Silah[3] and Bengi Uslu[1, 4]**

[1] Faculty of Pharmacy, Department of Analytical Chemistry, Ankara University, 06560, Ankara, Türkiye
[2] The Graduate School of Health Sciences, Ankara University, 06110, Ankara
[3] Faculty of Science, Department of Chemistry, Türkiye Bilecik Seyh Edebali University, 11210, Bilecik, Türkiye
[4]Integrated Research Center (BUTAM), Ankara University, Yapracik, 06790, Etimesgut,

Ankara, Turkiye

Correspondence should be addressed to Bengi Uslu*; buslu@pharmacy.ankara.edu.tr

KEYWORDS: Neratinib, BDDE, surfactant, voltammetry, Tween 20, serum

SUPPLEMENTAL MATERIALS

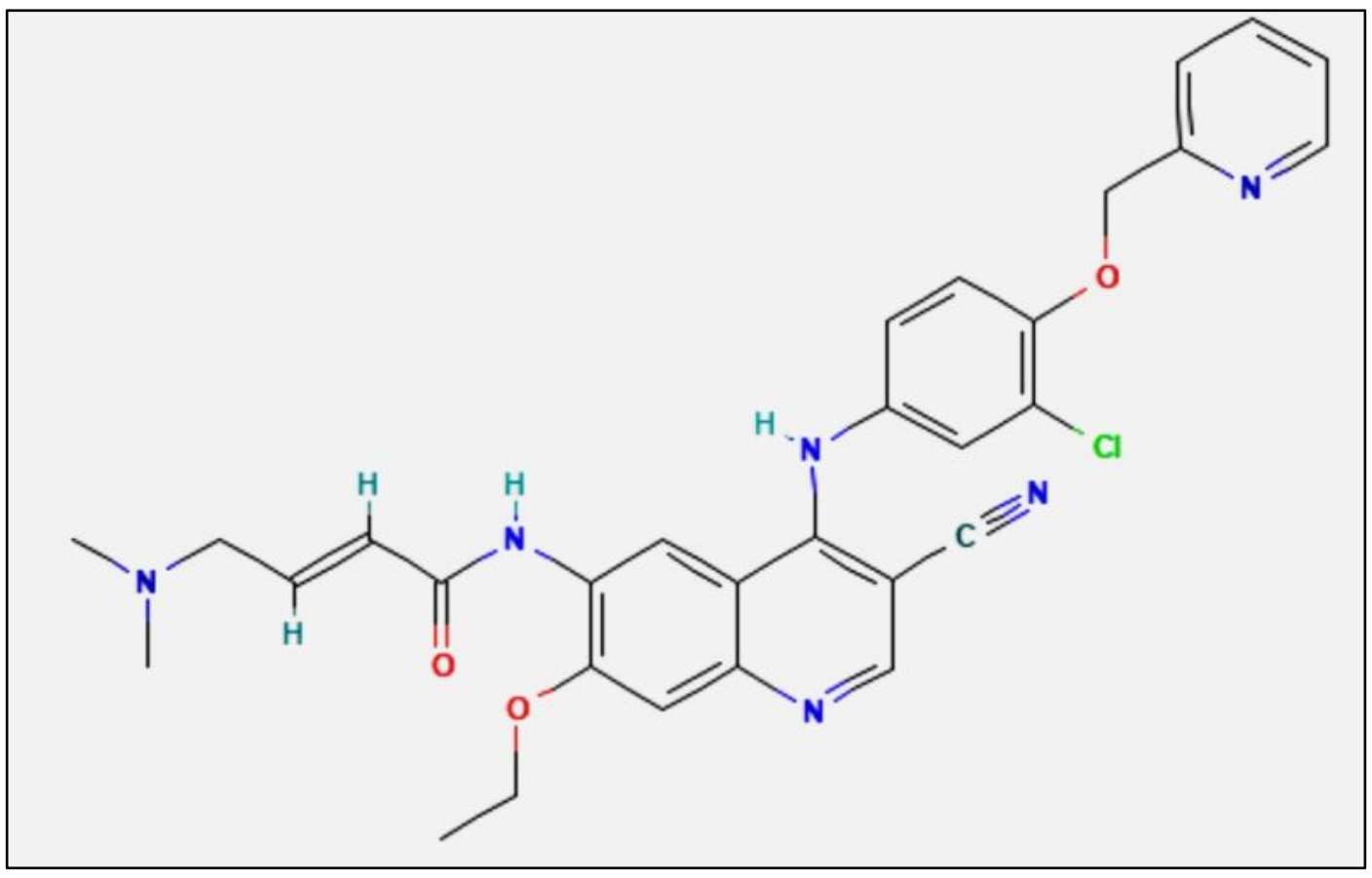

Fig. S1. Chemical structure of NER

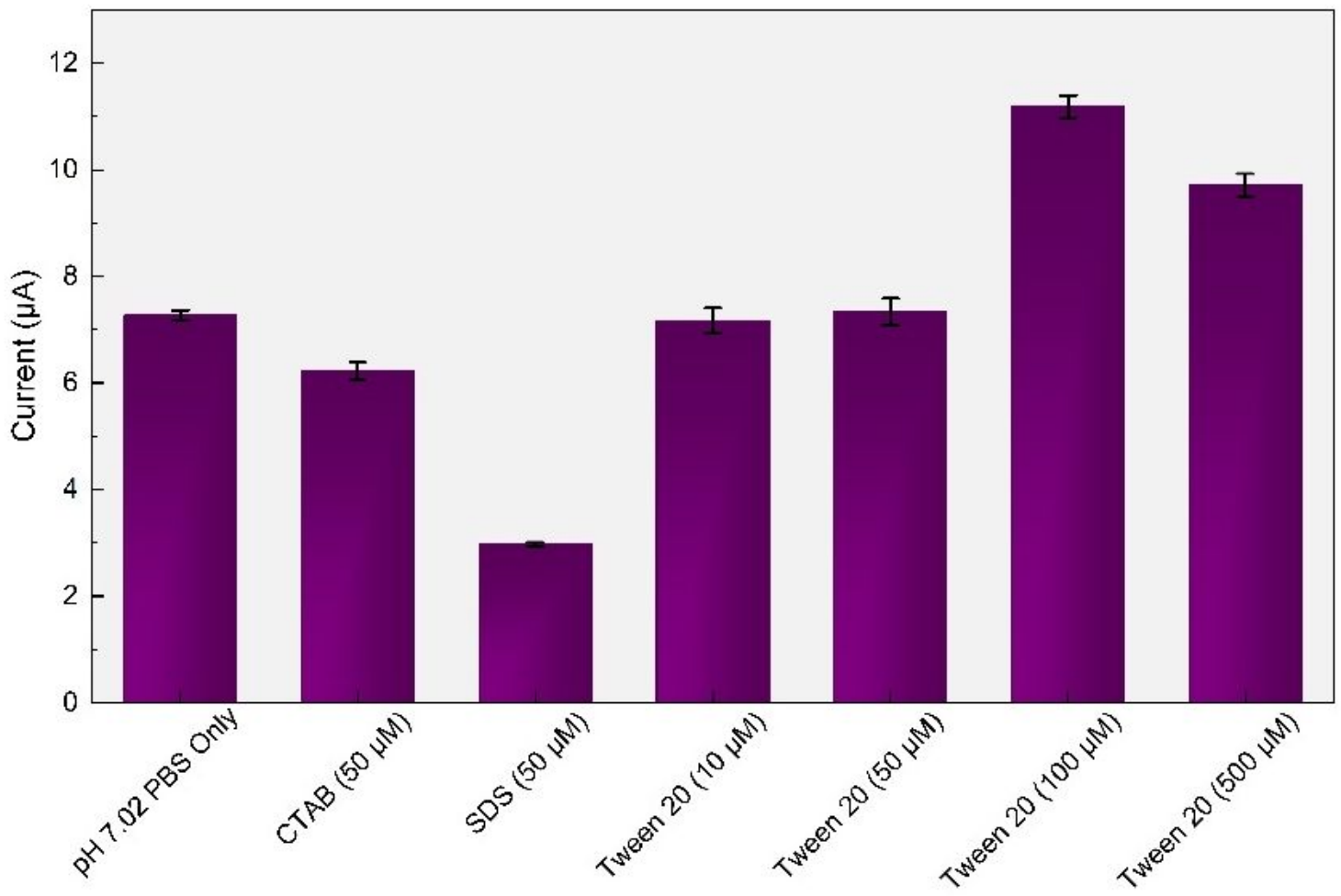

Fig. S2. Comparison of the anodic peak currents of 50 µM NER at BDDE in the absence and presence of different surfactants

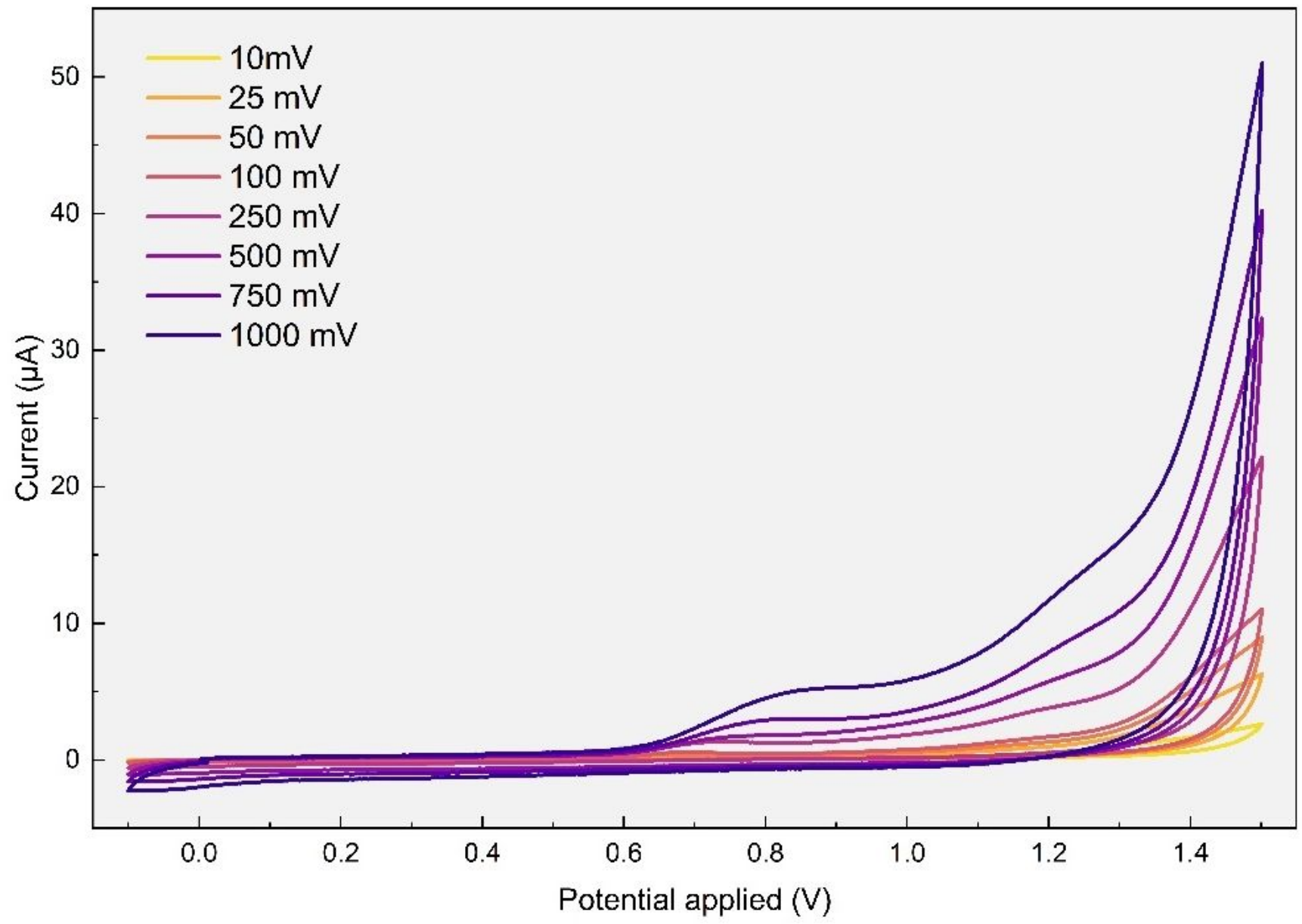

Fig. S3. Cyclic voltammograms of 0.50 μM NER in pH 7.02 PBS at BDDE over the scan rate range of 10-1000 mV/s.

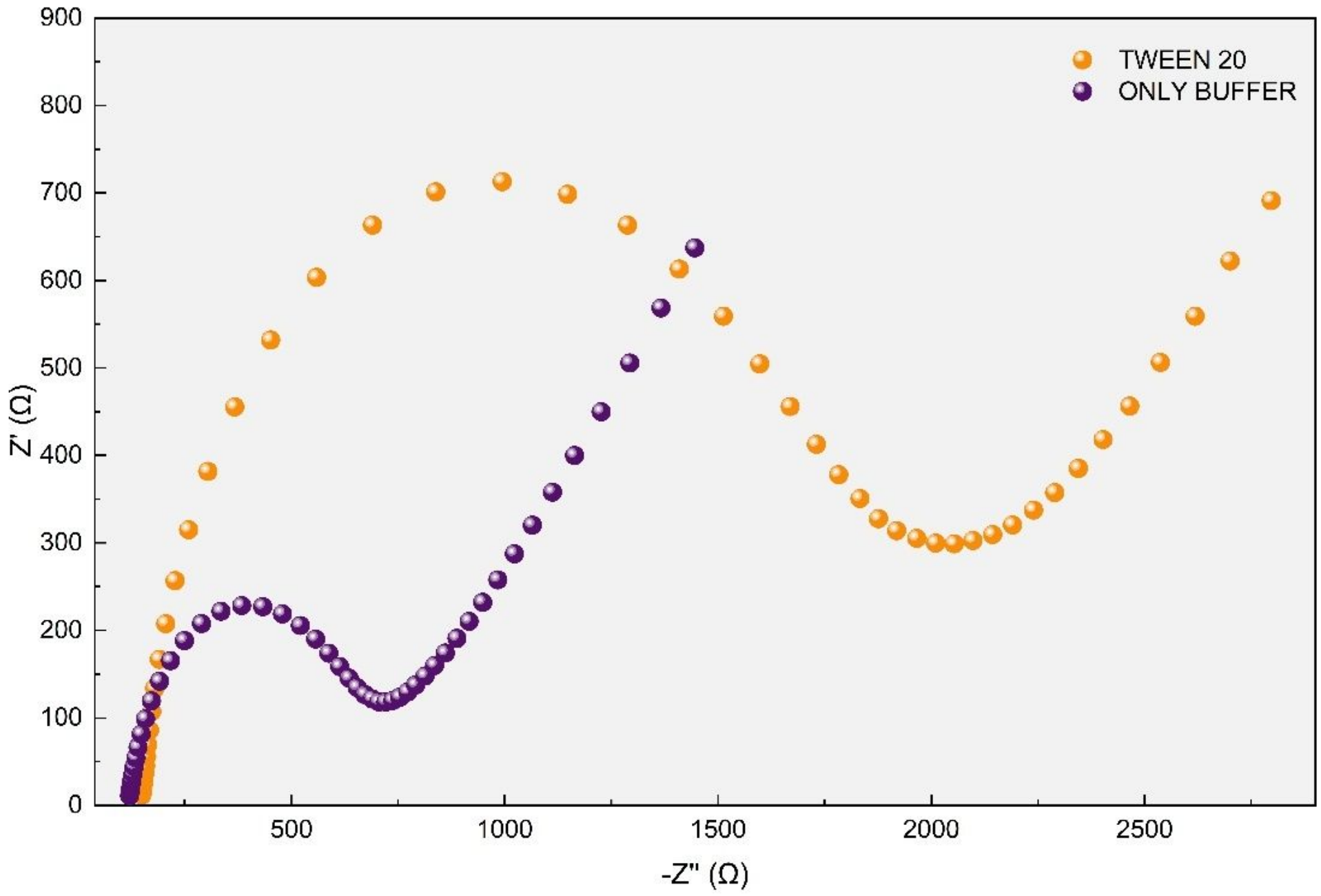

Fig. S4. Electrochemical characterization of the bare BDDE in pH 7.02 PBS buffer and Tween 20 solution utilizing EIS.

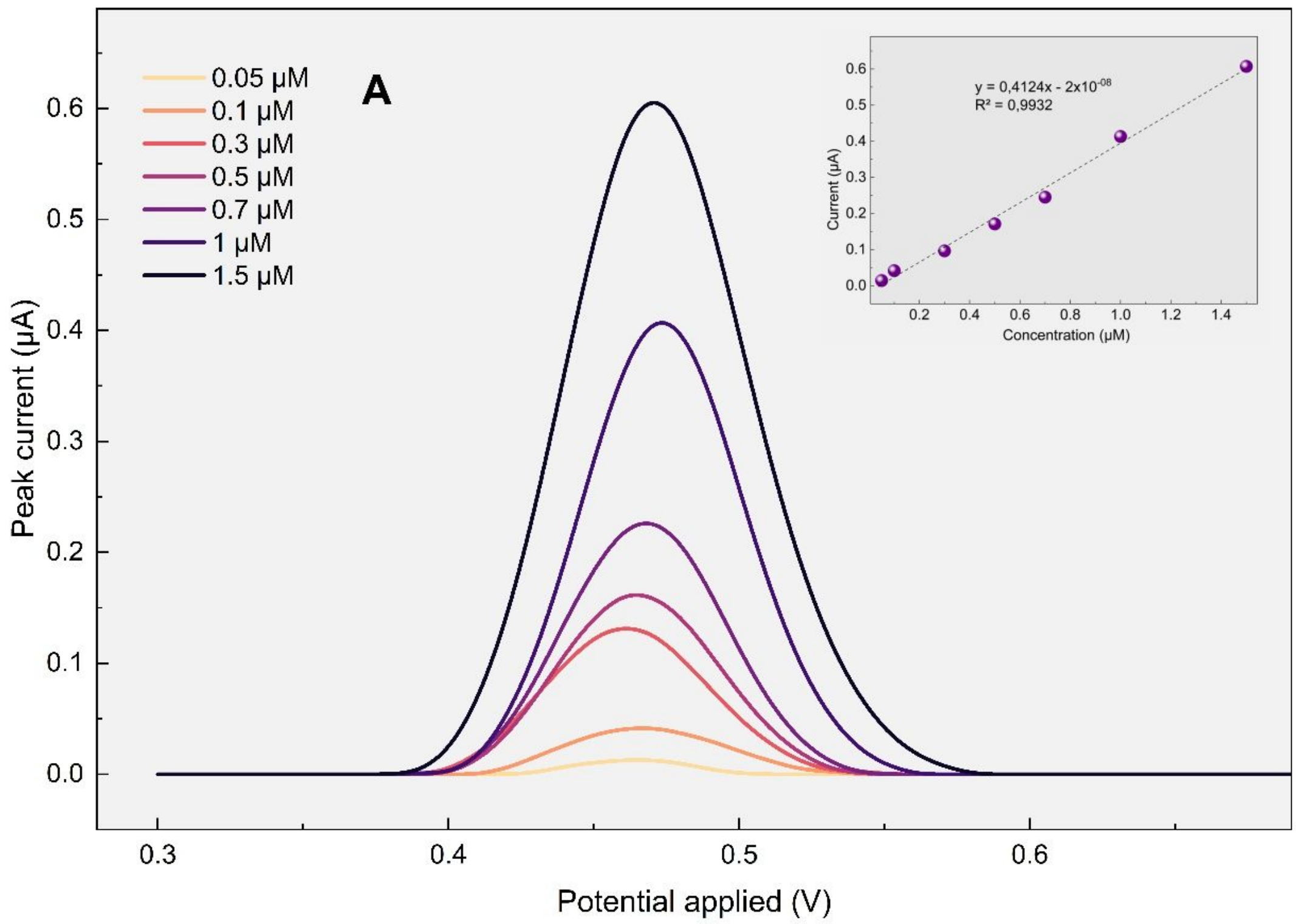

Fig. S5. DPVs of increasing concentrations of NER at BDDE in commercial human serum sample at pH 7.02 with 100 µM Tween 20. **(inset)** A graph of peak response versus NER concentration.

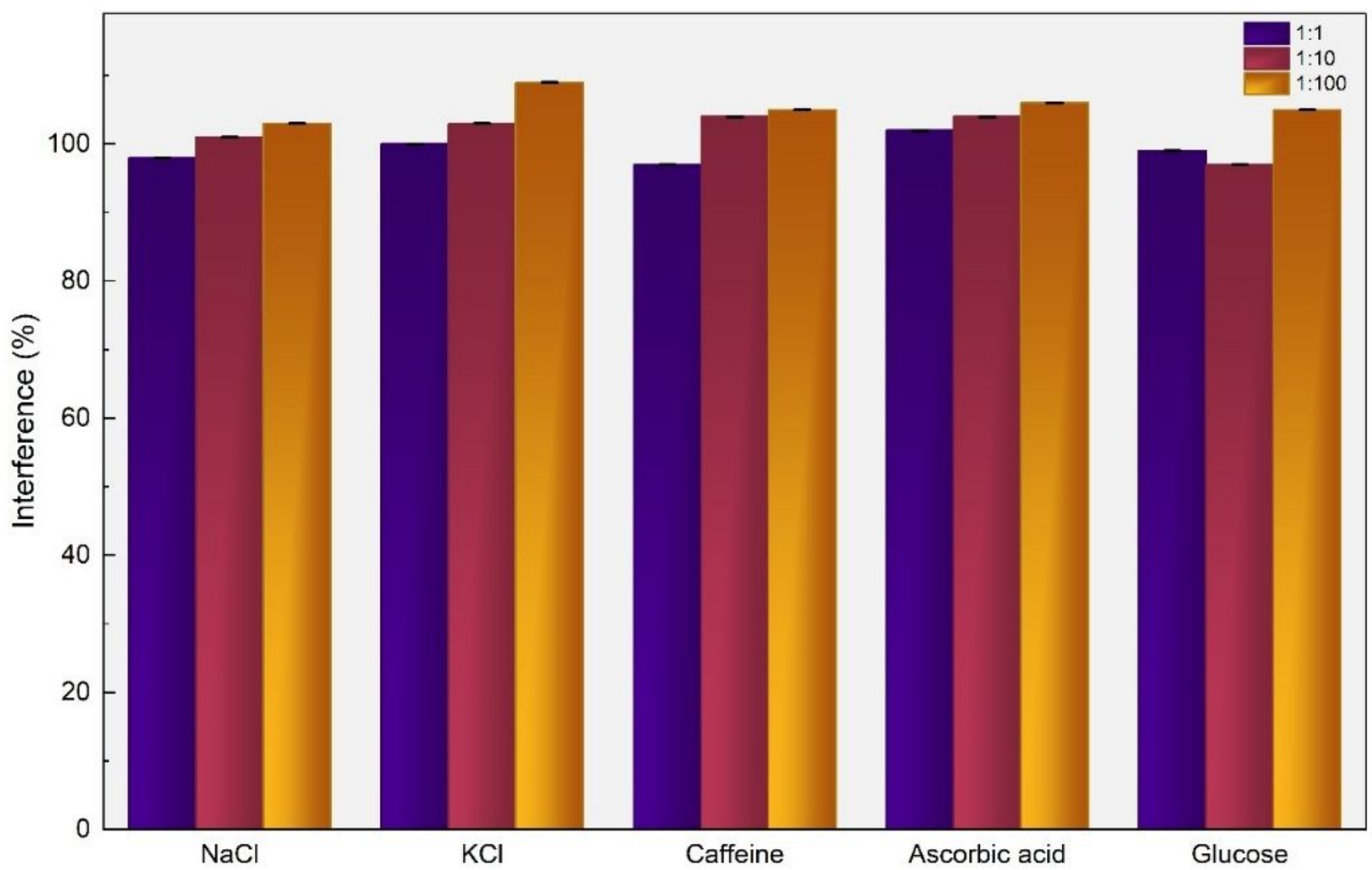

Fig. S6. Bar graphics give a demonstration of the influences of assorted concentrations of selected interfering species on the peak current of NER at BDDE under determined optimal experimental conditions.
